# Annual body mass index gain and risk of hypertensive disorders of pregnancy in a subsequent pregnancy

**DOI:** 10.1038/s41598-021-01976-y

**Published:** 2021-11-18

**Authors:** Sho Tano, Tomomi Kotani, Takafumi Ushida, Masato Yoshihara, Kenji Imai, Tomoko Nakano-Kobayashi, Yoshinori Moriyama, Yukako Iitani, Fumie Kinoshita, Shigeru Yoshida, Mamoru Yamashita, Yasuyuki Kishigami, Hidenori Oguchi, Hiroaki Kajiyama

**Affiliations:** 1 Department of Obstetrics and Gynecology, Nagoya University Graduate School of Medicine, Nagoya, Aichi Japan; 2 Department of Obstetrics, Perinatal Medical Center, TOYOTA Memorial Hospital, Toyota, Aichi Japan; 3 Division of Perinatology, Centre for Maternal-Neonatal Care, Nagoya University Hospital, Nagoya, Aichi Japan; 4 Department of Obstetrics and Gynecology, Fujita Health University School of Medicine, Toyoake, Aichi Japan; 5 Data Science Division, Data Coordinating Center, Department of Advanced Medicine, Nagoya University Hospital, Nagoya, Aichi Japan; 6 Kishokai Medical Corporation, Nagoya, Aichi Japan

**Keywords:** Health care, Medical research, Risk factors

## Abstract

Weight gain during interpregnancy period is related to hypertensive disorders of pregnancy (HDP). However, in interpregnancy care/counseling, the unpredictability of the timing of the next conception and the difficulties in preventing age-related body weight gain must be considered while setting weight management goals. Therefore, we suggest considering the annual change in the body mass index (BMI). This study aimed to clarify the association between annual BMI changes during the interpregnancy period and HDP risk in subsequent pregnancies. A multicenter retrospective study of data from 2009 to 2019 examined the adjusted odds ratio (aOR) of HDP in subsequent pregnancies. The aORs in several annual BMI change categories were also calculated in the subgroups classified by HDP occurrence in the index pregnancy. This study included 1,746 pregnant women. A history of HDP (aOR, 16.76; 95% confidence interval [CI], 9.62 − 29.22), and annual BMI gain (aOR, 2.30; 95% CI, 1.76 − 3.01) were independent risk factors for HDP in subsequent pregnancies. An annual BMI increase of ≥ 1.0 kg/m^2^/year was related to HDP development in subsequent pregnancies for women without a history of HDP. This study provides data as a basis for interpregnancy care/counseling, but further research is necessary to validate our findings and confirm this relationship.

## Introduction

Hypertensive disorders of pregnancy (HDP) are a group of syndromes defined by the onset of hypertension during pregnancy with an incidence of 8–10%^1^. The recurrence rate of HDP is as high as 20–60% in a subsequent pregnancy^2–5^. Women with a history of HDP are at an increased risk of future cardiovascular disease (CVD) and mortality^6–8^, which is further increased in women with recurrent events compared to those with a single event^9,10^. Therefore, although HDP shows spontaneous postpartum remission, it affects the outcomes of subsequent pregnancies and women’s health later in life. Thus, there is an urgent need to establish a strategy to prevent HDP in subsequent pregnancies.

Interpregnancy care/counseling has recently been recognized for its beneficial role in maternal health and subsequent pregnancy outcomes^11–13^. In addition to a history of HDP and being overweight/obese (BMI ≥ 25.0 kg/m^2^), interpregnancy body mass index (BMI) gain, defined as the difference between the pre-pregnant BMI of the index pregnancy and that of the subsequent pregnancy, is reported to be associated with HDP^3,14–17^. The American College of Obstetricians and Gynecologists (ACOG) recommends body weight management during the interpregnancy period to reduce the recurrence risk of HDP; however, no clear goal has been stated to this effect^11^. The total BMI gain during the interpregnancy period is certainly a valuable indicator for detecting high-risk for HDP at the first visit for subsequent pregnancy; however, the metric has no role or relevance in the prevention of HDP in subsequent pregnancies at the interpregnancy care/counseling stage, which is provided just after childbirth (index pregnancy). Most women do not plan when they will have another baby, and it is difficult to plan a weight management goal. We should also consider the difficulty in preventing age-related weight gain, as reported previously^18^. Longitudinal studies have reported that approximately 0.5 kg is the mean spontaneous age-related annual weight gain (natural gain) in women younger than 50 years^19,20^. For Japanese women of average height (157.9 cm), approximately 0.2 kg/m^2^/year is implied as the spontaneous age-related annual BMI gain. Considering the unpredictability of the timing of next conception and the difficulty of compensation for age-related weight gain, goal setting based on the total change in BMI during the interpregnancy period can be ambiguous. This process of goal setting must include goals with a clear timeframe and allow for some weight gain in these women.

It is generally essential to consider the target population, attainability, and the timeframe when formulating these goals^21^. To address these issues, the concept of “annual BMI change” was applied in the present study to provide a standard for body weight management in interpregnancy care/counseling; annual BMI change would be preferable to total BMI change as the former would help set more realistic goals with an explicit timeframe. Moreover, “annual BMI change” has already been beneficial in various medical and healthcare fields, including those related to oncology^22,23^, CVD risks^24,25^, severe obstructive sleep apnea^26^, and diabetes mellitus^27,28^. However, no report has reported the association between the annual BMI change and HDP risk. Thus, it is necessary to evaluate whether an annual BMI gain of 0.2 (natural gain) or more kg/m^2^/year is related to HDP occurrence in the subsequent pregnancy.

This study aimed to examine the association between annual BMI changes during the interpregnancy period and HDP in a subsequent pregnancy. Furthermore, we also aimed to conduct several subgroup analyses considering the adequate annual BMI change, as we believe that it would be help in the interpregnancy care/counseling based on individual characteristics, including pre-pregnant BMI and the outcome of the index pregnancy.

## Methods

### Study population

**Figure 1:**
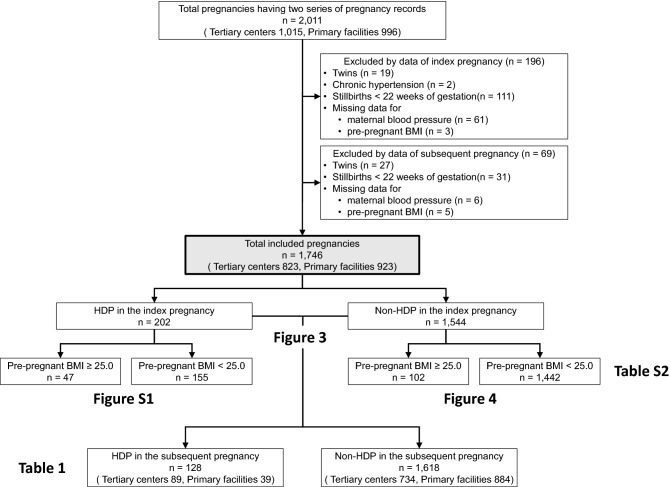
Flow chart of the study subjects. Clinical data were obtained from 2011 women who delivered at two tertiary centers or 12 primary maternity care units for whom two serial pregnancy records were available were obtained. A total of 1746 women were eligible for this study after excluding 196 and 69 women based on the index and subsequent pregnancy data, respectively.

This multicenter retrospective study used data obtained from an electronic medical records system. Data were collected for women aged 15 years or older who delivered at Nagoya University Hospital or TOYOTA Memorial Hospital, Aichi prefecture, or 12 private maternity facilities (Kishokai Medical Corporation located in Aichi and Gifu prefectures) in 2009–2019. We assessed the records directly and ascertained the data, including blood pressure if necessary. Women whose medical records for the index and the subsequent pregnancy could be obtained were included. The exclusion criteria were as follows: multiple pregnancy, stillbirth before 22 weeks of gestation, and data missing for maternal blood pressure and pre-pregnant BMI (Fig. 1). Women with chronic hypertension were also excluded. Since all patients with chronic hypertension are diagnosed with HDP at the point of conception^1^, including them in the study population was inappropriate for evaluating the risk of HDP development after conception. Women who developed HDP in a subsequent pregnancy were included in the HDP group, while those who did not were included in the non-HDP group. HDP was diagnosed according to the definitions proposed by the International Society for the Study of Hypertension in Pregnancy in 2018^1^, but chronic hypertension was excluded for the abovementioned reasons. Thus, the HDP group included only women with gestational hypertension and preeclampsia.

This study was approved by the Nagoya University Hospital ethics committee (approval number: 2015–0415) and all research was performed in accordance with relevant guidelines/regulations and the Declaration of Helsinki. The requirement for informed consent was waived by the Nagoya University Hospital ethics committee because of the retrospective nature of the study.

### Definitions of variables

**Figure 2:**
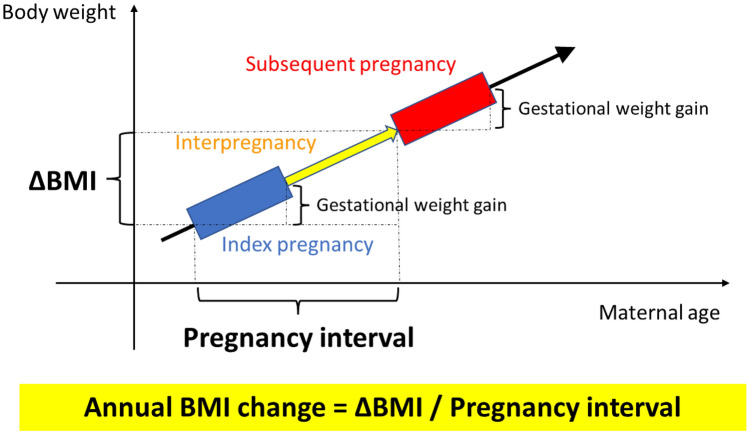
Overview of the definitions of terms. We defined interpregnancy BMI change (ΔBMI) as a change in pre-pregnant BMI from the index pregnancy to the subsequent pregnancy. The interpregnancy interval was defined as the interval from the expected date of delivery of the index pregnancy to that of the subsequent pregnancy, which is equivalent to the interval between the two conceptions. The annual BMI change was calculated as the ΔBMI/pregnancy interval. Gestational weight gain was defined as the change between pre-pregnant body weight and that before delivery. BMI, body mass index.

We used the self-reported maternal height and pre-pregnant body weight obtained during routine practice to calculate BMI (kg/m^2^) (weight in kg divided by squared height in m^2^). The subjects were categorized as underweight (< 18.5 kg/m^2^), normal weight (18.5–24.9 kg/m^2^), overweight (25.0–29.9 kg/m^2^), or obese (≥ 30.0 kg/m^2^) as reported by the World Health Organization consultation^29^. For the subgroup analyses, the participants were dichotomized on the basis of pre-pregnant BMI (< 25.0 or ≥ 25.0 kg/m^2^) in the index pregnancy^30^. Women with prepregnancy diabetes mellitus (DM) or hemoglobin A1_C_ level ≥ 6.5% (48 mmol/mol) or fasting plasma glucose level ≥ 126 mg/dL during pregnancy were defined as having overt DM. Assisted reproductive technology (ART) was defined as conception after in vitro fertilization or intracytoplasmic sperm injection. Gestational weight gain was defined as the difference between pre-pregnant body weight and the weight of the woman before delivery. Gestational age (GA) was routinely assessed by the expected date of delivery (EDD) determined as based on the last menstruation period and the measurement of the crown-rump length by ultrasonography. In ART pregnancies, EDD was determined based on the age of the embryo and the date of transfer. Gestational DM (GDM) was diagnosed according to oral glucose tolerance test results of fasting plasma glucose level ≥ 92 mg/dL or 1-h and 2-h plasma glucose level after 75-g glucose loading of ≥ 180 mg/dL or ≥ 153 mg/dL, respectively. Light for date was diagnosed using the Japanese standards for birth weight according to pregnancy duration^31^. As shown in Fig. 2, we defined interpregnancy BMI change (ΔBMI) as a change in pre-pregnant BMI from the index pregnancy to the subsequent pregnancy, as previously reported^14,30^. Interpregnancy interval was defined as the interval from the EDD of the index pregnancy to that of the subsequent pregnancy, which is equivalent to the interval between the two conceptions. The annual BMI change was calculated as the ΔBMI/pregnancy interval. Annual BMI change during the interpregnancy period was categorized into five groups (< 0.0 [weight loss], ≥ 0.0– < 0.2 [reference, natural gain], ≥ 0.2– < 0.6, ≥ 0.6– < 1.0 and ≥ 1.0 kg/m^2^/year), since an annual gain of 0.2 kg/m^2^/year is considered normal and unavoidable^19,20^; 0.6 and 1.0 kg/m^2^/year gains are equivalent to approximately 1.5 and 2.5 kg/year increases in the weight of women of average height (157.9 cm), respectively. Comparisons were performed among these categories accordingly to establish body weight management goals keeping in mind the normal range of weight gain.

### Statistical analysis

Clinical characteristics and parameters (Table 1) of the HDP and non-HDP groups were compared using the χ^2^ test, Fisher’s exact test, Student’s *t*-test, Welch’s *t*-test, or the Mann–Whitney U test as appropriate. Crude and adjusted odds ratios (aORs) were calculated for HDP in the subsequent pregnancy using univariable and multivariable logistic regression analyses, respectively. Variables pertaining to the index pregnancy or interpregnancy period, which are known to be associated with HDP in subsequent pregnancies, were selected for analysis. In the multivariable logistic regression analysis, backward elimination methods were used to identify factors associated with HDP in the subsequent pregnancy, and variables with values of *p* < 0.25 in the univariable logistic regression analysis were entered in the multivariable logistic regression analysis. Multivariable logistic regression analysis of well-known risk factors for HDP^3,14–17^ was also performed for sensitivity analysis. We classified annual BMI gain into four or five categories based on their distributions as mentioned above and performed a multivariable analysis to determine how the aOR changed with a specific annual BMI change.

**Table 1:** Baseline characteristics and perinatal outcomes.

	HDP in the subsequent pregnancy	Non-HDP in the subsequent pregnancy	*p* Value
	n = 128	n = 1,618	
Tertiary center	89 (69.5)	734 (45.4)	< 0.001*
**Index pregnancy**			
Maternal age, years old	31.3 ± 4.8	30.4 ± 4.8	0.054
Pre-pregnant BMI	24.3 ± 5.4	20.6 ± 2.9	< 0.001*
Overweight/Obesity (BMI ≥ 25.0)	47 (36.7)	102 (6.3)	< 0.001*
Smokers	0 (0.0)	17 (1.1)	1.000
OvertDM	4 (3.1)	14 (0.9)	0.055
Hyperthyroidism	1 (0.8)	15 (0.9)	1.000
Hypothyroidism	3 (2.3)	32 (2.0)	1.000
Primiparity	96 (75.0)	1,111 (68.7)	0.596
ART	20 (15.6)	124 (7.7)	0.001*
Gestational body weight gain, kg	11.5 ± 4.8	10.9 ± 3.8	0.149
HDP	72 (56.3)	130 (8.0)	< 0.001*
GA at the onset of HDP, weeks, median [p25, p75]	32.9 [27.2, 37.3]	36.7 [30.8, 38.7]	0.005*
Early-onset HDP	39/72 (54.2)	41/130 (31.5)	0.002*
PE	17/72 (23.6)	37/130 (28.5)	0.456
GDM	11 (8.6)	55 (3.4)	0.005*
Stillbirth > 22 weeks of gestation	1 (0.8)	13 (0.8)	1.000
GA at delivery, weeks	39.0 ± 2.1	39.1 ± 2.1	0.490
Preterm birth (< 37 weeks of gestation)	15 (11.7)	118 (7.3)	0.069
Cesarean section	39 (30.5)	376 (23.2)	0.066
Neonatal sex, male	59 (46.1)	883 (54.6)	0.064
Neonatal height, cm	49.2 ± 3.2	49.4 ± 2.9	0.428
Birthweight, g	2,970 ± 553	2,976 ± 494	0.891
Light for date infant	9 (7.0)	124 (7.7)	0.683
Placental weight, g	580.2 ± 125.6	571.1 ± 112.7	0.390
**Inter-pregnancy**			
Pregnancy interval, years, median [p25, p75]	2.1 [1.6, 2.8]	2.0 [1.7, 2.5]	0.317
ΔBMI	0.99 ± 1.88	0.40 ± 1.37	0.001*
Anuual BMI change, kg/m^2^/year	0.60 ± 1.42	0.19 ± 0.77	0.002*
**Subsequent pregnancy**			
Maternal age, years old	33.7 ± 5.0	32.7 ± 5.0	0.017*
Pre-pregnant BMI	25.3 ± 5.7	21.0 ± 3.0	< 0.001*
Overweight (BMI > 25.0)	57 (44.5)	137 (8.5)	< 0.001*
Smokers	0 (0.0)	17 (1.1)	1.000
OvertDM	5 (3.9)	16 (1.0)	0.009*
Hyperthyroidism	1 (0.8)	15 (0.9)	1.000
Hypothyroidism	3 (2.3)	32 (2.0)	1.000
ART	18 (14.1)	122 (7.5)	0.007*
Gestational body weight gain, kg	10.3 ± 4.2	10.2 ± 3.6	0.688
HDP	128 (100)	0 (0.0)	–
GA at the onset of HDP, weeks, median [p25, p75]	36.5 [31.4, 38.0]	–	–
Early-onset HDP	44 (34.4)	–	–
PE	30 (23.4)	–	–
GDM	20 (15.6)	121 (7.5)	0.003*
Stillbirth ≥ 22 weeks of gestation	0 (0.0)	1 (0.1)	1.000
GA at delivery, weeks	38.8 ± 1.9	39.0 ± 1.5	0.141
Preterm birth (< 37 weeks of gestation)	11 (8.6)	79 (4.9)	0.068
Cesarean section	41 (32.0)	371 (22.9)	0.020*
Neonatal sex, male	75 (58.6)	818 (50.6)	0.076
Neonatal height, cm	49.7 ± 3.4	49.8 ± 2.1	0.936
Birthweight, g	3,026 ± 601	3,054 ± 405	0.599
Light for date infant	12 (9.4)	49 (3.0)	0.002*
Placental weight, g	600.9 ± 143.2	581.1 ± 108.9	0.127

HDP, hypertensive disorders of pregnancy; BMI, body mass index; DM, diabetes mellitus; ART, assisted reproductive technology; GA, gestational age; PE, preeclampsia; GDM, gestational diabetes mellitus.

Data are presented as means ± standard deviation or median [p25, p75] for continuous variables and n (%) for discrete variables.

*Statistically significant.

Data are presented as mean ± standard deviation or median [p25, p75] for continuous variables and number (percentage) for categorical variables. Statistical significance was set at *p* < 0.05. The statistical analyses were conducted using SPSS version 27.0 for Windows software (SPSS, Inc., Chicago, IL, USA).

## Results

### Participants

A total of 2,011 pregnant women (tertiary centers, n = 1,015; primary maternity care units, n = 996) were included. Among them, 206 women were excluded due to multiple pregnancies (n = 19 and 27), chronic hypertension (n = 2 and 0), stillbirth before 22 weeks of gestation (n = 111 and 31), and missing data for maternal blood pressure (n = 61 and 6) or pre-pregnant BMI (n = 3 and 5) in the index and subsequent pregnancy, respectively (Fig. 1). The remaining 1,746 pregnant women (tertiary centers, n = 823; primary maternity care units, n = 923) were finally included.

### Comparison of clinical parameters between the HDP and non-HDP groups

HDP occurred in a subsequent pregnancy in 128/1,746 (7.3%) participants, 69.5% of whom were treated at tertiary centers. Table 1 presents data of women who developed HDP in the subsequent pregnancy (HDP group) and those who did not develop HDP in the subsequent pregnancy (non-HDP group).

With regard to the variables of the index pregnancy (HDP group vs. non-HDP group), there were no significant differences in maternal age (31.3 ± 4.8 vs. 30.4 ± 4.8 years, *p* = 0.054), the incidence of smoking between the two groups (0.0 vs. 1.1%, *p* = 1.000). Pre-pregnant BMI (24.3 ± 5.4 vs. 20.6 ± 2.9 kg/m^2^, *p* < 0.001) and the incidence of HDP (56.3 vs. 8.0%; *p* < 0.001) were significantly higher in HDP group. The median GA at the onset of HDP was lower in the HDP group than in the non-HDP group (median [p25-p75]; 32.9 [27.2–37.3] vs. 36.7 [30.3–38.7] weeks,* p* = 0.005); therefore, the HDP group included more patients with early-onset HDP than those in the non-HDP group (54.2 vs. 31.5%, *p* = 0.002). The incidence of conception by ART and GDM was also higher in the HDP group than in the non-HDP group (15.6 vs. 7.7%,* p* = 0.001 and 8.6 vs. 3.4%, *p* = 0.005, respectively).

Regarding the variables pertaining to the interpregnancy period (HDP group vs. non-HDP group), the pregnancy interval did not differ significantly between the two groups (median [p25-p75]; 2.1 [1.6–2.8] vs. 2.0 [1.7–2.5] years,* p* = 0.317), whereas the ΔBMI and annual BMI change were significantly higher in the HDP group than in the non-HDP group (0.99 ± 1.88 vs. 0.40 ± 1.37 kg/m^2^, *p* = 0.001 and 0.60 ± 1.42 vs. 0.19 ± 0.77 kg/m^2^/year, *p* = 0.002, respectively).

With regard to the variables pertaining to the subsequent pregnancy (HDP group vs. non-HDP group), the incidence of conception by ART and GDM was significantly higher in the HDP group than in the non-HDP group (14.1% vs. 7.5%,* p* = 0.007 and 15.6% vs. 7.5%, *p* = 0.003, respectively). The mean pre-pregnant BMI was also higher in the HDP group than in the non-HDP group (25.3 ± 5.7 vs. 21.0 ± 3.0 kg/m^2^, *p* < 0.001). Regarding the perinatal outcomes of the subsequent pregnancy, the Cesarean section and light for date rates were significantly higher in the HDP group than in the non-HDP group (32.0% vs. 22.9%,* p* = 0.020 and 9.4% vs. 3.0%, *p* = 0.002, respectively).

### Risk factors for HDP in the subsequent pregnancy

Based on the results of univariable and multivariable analyses performed using the backward elimination method (Table 2), five variables remained in the final set, and the history of HDP (HDP development in the index pregnancy) showed the highest aOR for HDP in the subsequent pregnancy (aOR, 16.76; 95% confidence interval [CI], 9.62–29.22), followed by annual BMI gain in the interpregnancy period (aOR, 2.30; 95% CI, 1.76–3.01), pre-pregnant BMI in the index pregnancy (aOR, 1.25; 95% CI, 1.17–1.33), and maternal age in the index pregnancy (aOR, 1.07; 95% CI, 1.01–1.13). Those results were also similar after controlling simultaneously for potential confounding variables: maternal age, pre-pregnant BMI, gestational body weight gain, overt DM, ART, HDP, GDM in the index pregnancy; interpregnancy period, and annual BMI change during the interpregnancy period (Table S1, Model 1). Furthermore, the results were similar even after adjusting for the tertiary centers (Table S1, Model 2).

**Table 2:** Univariable and multivariable logistic regression analysis of factors potentially associated with HDP in a subsequent pregnancy.

	Univariable analysis			Multivariable analysis		
	OR	95%CI	*p* Value	aOR	95%CI	*p* Value
**Index pregnancy**						
Maternal age^†^, years old	1.04	(1.00–1.08)	0.054	1.07	(1.01–1.13)	0.021*
Pre-pregnant BMI^†^, kg/m^2^	1.25	(1.20–1.30)	< 0.001	1.25	(1.17–1.33)	< 0.001*
Primiparity^†^	1.14	(0.70–1.84)	0.596			
Gestational body weight gain^†^, kg	1.04	(1.00–1.09)	0.077	–	–	–
Overt DM^†^	3.24	(1.05–10.01)	0.041	–	–	–
ART^†^	2.28	(1.37–3.82)	0.002	–	–	–
HDP^†^	14.72	(9.94–21.79)	< 0.001	16.76	(9.62–29.22)	< 0.001*
GDM^†^	2.53	(1.29–4.97)	0.007	–	–	–
Stillbirth ≥ 22 weeks of gestation^†^	0.91	(0.12–7.02)	0.928			
GA at delivery^†^, weeks	0.97	(0.90–1.05)	0.490			
Preterm birth (< 37 weeks of gestation)^†^	1.69	(0.95–2.99)	0.072	–	–	–
Cesarean section^†^	1.45	(0.98–2.14)	0.067	–	–	–
Neonatal sex^†^, male	0.71	(0.50–1.02)	0.065	–	–	–
Neonatal height^†^, cm	0.98	(0.93–1.03)	0.428			
Birthweight^†^, g	1.00	(1.00–1.00)	0.891			
Light for date infant^†^	0.86	(0.43–1.75)	0.683			
Placental weight^†^, g	1.00	(1.00–1.00)	0.390			
**Inter-pregnancy**						
Pregnancy interval, years	1.26	(1.08–1.48)	0.004	1.57	(1.25–1.97)	< 0.001*
Anuual BMI change, kg/m^2^/year	1.57	(1.31–1.87)	< 0.001	2.30	(1.76–3.01)	< 0.001*

HDP, hypertensive disorders of pregnancy; OR, odds ratio; CI, confidence interval; BMI, body mass index; DM, diabetes mellitus; ART, assisted reproductive technology; GA, gestational age; PE, preeclampsia; GDM, gestational diabetes mellitus.

^†^Parameteres of the index pregnancy.

*Statistically significant.

**Figure 3:**
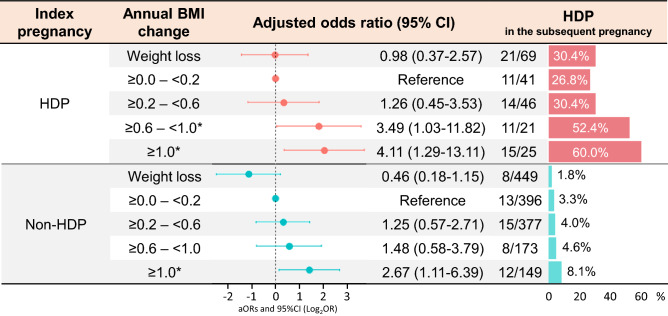
Adjusted odds ratios of classified annual BMI change for HDP in the subsequent pregnancy. The multivariable models were adjusted for maternal age in the index pregnancy, pre-pregnant BMI in the index pregnancy, classified annual BMI change, and interpregnancy interval. The values on the left side of the graph are expressed as log_2_OR. The right side of the graph shows the incidence of HDP in subsequent pregnancies according to the degree of annual BMI change. The number of HDP events in the subsequent pregnancy/total number is also shown according to the degree of annual BMI change. BMI, body mass index; CI, confidence interval; HDP, hypertensive disorders of pregnancy *Statistically significant.

The aORs of HDP in the subsequent pregnancy were also evaluated by classifying annual BMI changes into five categories distribution (Fig. 3). In the subgroup of HDP in the index pregnancy (Fig. 3, upper panel), the aORs in the 0.6–1.0 kg/m^2^/year gain and ≥ 1.0 kg/m^2^/year gain groups were approximately 3.49 (95% CI, 1.03–11.82) and 4.11 (95% CI, 1.29–13.11), respectively. However, in the subgroup of non-HDP in the index pregnancy (Fig. 3, lower panel), the aOR was significantly increased only in the ≥ 1.0 kg/m^2^/year gain (aOR, 2.67; 95% CI, 1.11–6.39).

**Figure 4:**
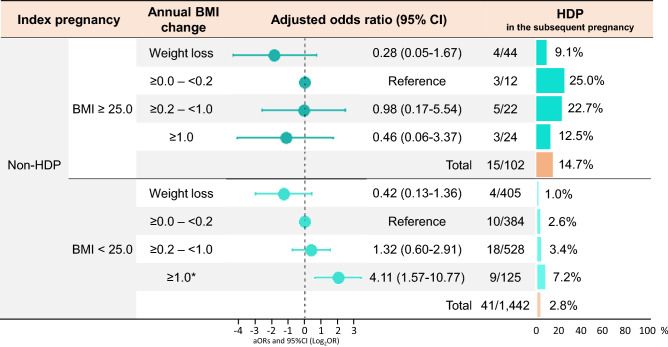
Adjusted odds ratios for HDP in the subsequent pregnancy among those without the history of HDP in the index pregnancy. The multivariable models were adjusted for maternal age in the index pregnancy, pregnancy interval, and classified annual BMI change. Values on the left side of the graph are expressed as log_2_OR. The right side of the graph shows the incidence of HDP in the subsequent pregnancy according to the degree of annual BMI change. The number of HDP in the subsequent pregnancy/total number is also shown according to the degree of annual BMI change. BMI, body mass index; CI, confidence interval; HDP, hypertensive disorders in pregnancy.

We further analyzed for women without a history of HDP, who were divided according to pre-pregnant BMI (≥ or < 25.0 kg/m^2^) in the index pregnancy (Fig. 4) since the recurrent risk was high in women with a history of HDP. The annual BMI change during the interpregnancy period was categorized into four groups (< 0.0 [weight loss], ≥ 0.0– < 0.2 [reference], ≥ 0.2– < 1.0, and ≥ 1.0 kg/m^2^/year) since the aORs of HDP in the subsequent pregnancy were increased in ≥ 1.0 kg/m^2^/year, respectively, in women without a history of HDP (Fig. 3, lower panel). Among patients without a history of HDP, the prevalence of HDP in the subsequent pregnancy was higher in women with pre-pregnant BMI of ≥ 25.0 kg/m^2^ in the index pregnancy (15/102, 14.7%) than in those with pre-pregnant BMI of < 25.0 kg/m^2^ in the index pregnancy (41/1,442, 2.8%). In women with pre-pregnant BMI ≥ 25.0 kg/m^2^, the prevalence of HDP was lower in the weight loss group than in the weight gain groups (4/44, 9.1% vs. 11/58, 19.0%), although the difference was not significant (aOR 0.28, 95%CI, 0.05–1.67) (Fig. 4, upper panel). In women with pre-pregnant BMI of < 25.0 kg/m^2^, the aOR for HDP in the subsequent pregnancy was significant only when they had an annual weight gain of ≥ 1.0 kg/m^2^/year (aOR, 4.11; 95% CI, 1.57–10.77) (Fig. 4, lower panel). Further, among the women with pre-pregnant BMI of < 25.0 kg/m^2^ and who did not develop HDP in the index pregnancy, those women who developed HDP in the subsequent pregnancy had significantly higher gestational weight gain in the index pregnancy and greater annual BMI change than those without HDP in the subsequent pregnancy, although there were no significant differences in maternal age and pregnancy interval (Table S2).

The HDP recurrence rate was as high as 93.3% in women who had a pre-pregnant BMI of ≥ 25.0 kg/m^2^ in the index pregnancy and who had an annual BMI gain ≥ 0.6 kg/m^2^/year in the interpregnancy period (Fig. S1, upper panel). In women with HDP occurrence in the index pregnancy, the results were similar among the two subgroups with pregnant BMI of ≥ and < 25.0 kg/m^2^ in the index pregnancy; aOR and the prevalence of HDP was the highest in women with annual BMI gain ≥ 0.6 kg/m^2^/year during the interpregnancy period.

## Discussion

This is the first study to evaluate the association between HDP in a subsequent pregnancy and annual BMI change during the interpregnancy period. The annual BMI gain during the interpregnancy period was significantly related to HDP in subsequent pregnancies. Maternal age, pre-pregnant BMI, HDP in the index pregnancy, and interpregnancy interval were also variables significantly associated with HDP in the subsequent pregnancy. Among these variables, a history of HDP in the index pregnancy was most strongly related to HDP in the subsequent pregnancy. Thus, annual BMI change were investigated in two subgroups: women with or without a history of HDP in the index pregnancy. For women without a history of HDP in the index pregnancy, an annual BMI gain of ≥ 1.0 kg/m^2^/year was associated with HDP in the subsequent pregnancy. Further, in the subgroup with pre-pregnant BMI < 25.0 in the index pregnancy, an annual BMI gain of ≥ 1.0 kg/m^2^/year was associated with HDP in the subsequent pregnancy, although it was not related to HDP in the subgroup with pre-pregnant BMI ≥ 25.0 in the index pregnancy. However, for women with a history of HDP in the index pregnancy, an annual BMI gain of ≥ 0.6 kg/m^2^/year was related to HDP in the subsequent pregnancy. These results suggest that in interpregnancy care/counseling, appropriate annual BMI goals is better to be provided to the women based on their characteristics, including the presence or absence of HDP in the index pregnancy.

Several previous studies suggested that women with a history of HDP are at higher risk for HDP in the subsequent pregnancy^2,3,5^. The present study showed an approximate recurrence rate of 35.6%, which is consistent with previous reports^2,3,5^. The recurrence rate is also affected by the other factors including maternal age, pre-pregnant BMI, and interpregnancy interval^3,5,14,15,32,33^. The present data also showed similar trends. Overall interpregnancy BMI gain was also reported to be related to HDP in the subsequent pregnancy^16,17^, which is consistent with the present data. The mean annual BMI change was approximately 0.2 kg/m^2^/year in this study population. An annual BMI gain of < 0.6 kg/m^2^/year was not associated with HDP recurrence in the subsequent pregnancy, although exceeding annual BMI gain ≥ 0.6 kg/m^2^/year was significant.

Interpregnancy care/counseling is now recommended to improve subsequent pregnancy outcomes and long-term health^11–13,34,35^. In the present study, BMI gain during the interpregnancy period was significantly associated with HDP in the subsequent pregnancy, whereas no significant difference was observed in gestational weight gain. Gestational weight gain has been reported to be associated with the development of HDP in that pregnancy^36^; however, it was not significantly associated with HDP in the subsequent pregnancy as determined in this study. Thus, the BMI gain during the interpregnancy period might be more critical.

Although there are currently no clear recommendations for women with a history of HDP, those with a history of preterm births are recommended to avoid short interpregnancy intervals^34^. Thus, we must gather additional evidence for interpregnancy care protocols to prevent HDP. A previous systematic review and meta-analysis reported that a significant interpregnancy weight gain increases the risk of GDM, preeclampsia, and large for gestational age births^16^. Another systematic review and meta-analysis reported that weight gain increases a risk of HDP^17^. The results of the present study support those of these systematic reviews and meta-analyses. But the present study is the first to demonstrate the association between annual BMI gain and the development of HDP in subsequent pregnancy in women with or without a history of HDP in the index pregnancy. These results would provide a standard for body weight management in interpregnancy care/counseling.

HDP is a complex syndrome involving several factors, including genetic variants^37,38^, a history of HDP, and a pre-pregnant BMI ﻿≥ 25.0. In interpregnancy care/counseling, women who have HDP in the index pregnancy should also be recommended to use aspirin during the subsequent pregnancy to prevent HDP^11^. Additionally, those with a pre-pregnant BMI of ≥ 25.0 might be informed that approximately 90% of them might develop HDP in the subsequent pregnancy if their annual BMI increase by ≥ 0.6 kg/m^2^/year during the interpregnancy period (Fig. S1). Aspirin would be unsuitable for women without a history of HDP in the index pregnancy to prevent HDP. Thus, the annual BMI change might be significant for these patients, and this was the focus of this study. Among those with pre-pregnant BMI < 25.0, an annual BMI gain of ≥ 1.0 kg/m^2^/year was associated with an approximately 4.1-fold increased risk of HDP in the subsequent pregnancy. Those with a pre-pregnant BMI of < 25.0, and no history of HDP in the index pregnancy are more likely to develop HDP in the subsequent pregnancy if they had high gestational weight gain in the index pregnancy and high annual BMI gain during the interpregnancy period, which would lead to overweight/obesity (BMI of ≥ 25.0) in the subsequent pregnancy (Table S2). This suggests that this population might be susceptible to weight gain. We are also going to explore whether weight gain would cause HDP in these low-risk women directly or indirectly.

However, among those with pre-pregnant BMI ≥ 25.0, annual BMI change was not associated with the risk of HDP in the subsequent pregnancy, but the prevalence of HDP in the subsequent pregnancy was not very low. Furthermore, women with weight loss during the interpregnancy period had a lower incidence of HDP in the subsequent pregnancy than women with annual BMI gain, although no significant difference was detected in this study. These results suggest that the appropriate standard of annual BMI change during the interpregnancy period might vary depending on the pre-pregnant BMI status and history of HDP in the index pregnancy. These findings would be helpful in interpregnancy care/counseling based on individual characteristics. However, further studies are needed to understand whether weight management using annual BMI scale is possible to prevent HDP in subsequent pregnancies.

Other studies reported that an interpregnancy interval of more than five years increased a risk of developing HDP in a subsequent pregnancy^33^. The present study also showed that a longer interpregnancy interval was an independent risk factor for HDP in the subsequent pregnancy; however, the interpregnancy interval exceeded five years in only 2.1% (36/1,746) of patients in this study.

### Strengths and limitations

This study has the following strengths. First, this is the first to assess the association between HDP in the subsequent pregnancy and the annual BMI changes during the interpregnancy period. Second, the association was reanalyzed by stratifying by other factors including a history of HDP and pre-pregnant BMI in the index pregnancy. Third, since this was a multicenter study, both tertiary care centers and primary maternity care units participated in this study. The study population included women with various risk levels, which could minimize selection bias. Previous studies of the recurrent risk of HDP included only women who gave birth at tertiary centers^14,15,39^. Furthermore, the data in this study were detailed and reliable as required by national registry studies.

This study has several limitations. First, only women for whom both index and subsequent pregnancy records were available were included. The following populations were not included: women who did not have a subsequent baby; and those who delivered a subsequent baby at a non-participating institute. Women with life-threatening complications, including brain hemorrhage, would not have a subsequent pregnancy and were also excluded. This impact is expected to be minimal because such severe cases are rare. However, we also could not include women who had an abortion in a subsequent pregnancy or those who developed infertility after the index pregnancy. These populations may have higher BMI gains than the study population, but this was not the focus of this study. The exclusion of individuals for missing data might have caused some bias. Those individuals were older and had a longer interpregnancy period, but other variables, including pre-pregnant BMI in the index pregnancy, ΔBMI, and annual BMI change, were not significantly different from those in this study population (data not shown). Therefore, the bias was minimal. Second, we did not follow up postpartum weight. Annual BMI gain was not measured as an annual check, but it was calculated according to ΔBMI and interpregnancy interval. However, these values would be correlated with each other because the mean interpregnancy interval was 2.3 years. Weight gain from prepregnancy to 18 months postpartum was recently reported as related to the subsequent risk of CVD in Danish women^40^. Interpregnancy health checkups for women who hope to have subsequent pregnancies have not yet been implemented in the clinical setting in Japan, but we plan to do so based on the present study's findings. Additionally, self-reported weight was used to calculate BMI in this study. However, most participants measured their weights at the prenatal visit in the first trimester, so the difference between the self-reported and actual values is likely to be minimal. Third, the analysis did not include maternal medication as a variable. Although aspirin use is one of the strategies to reduce the risk of HDP recurrence^11^, aspirin has not been used to prevent recurrent HDP in Japan. Thus, although we did not collect information on medications, we presume that only a few participants of this study would have used aspirin.

## Conclusion

In this study, a history of HDP was most strongly associated with HDP in a subsequent pregnancy. Furthermore, an annual weight gain during the interpregnancy period of ≥ 0.6 kg/m^2^/year was related to HDP recurrence in women with a history of HDP. For women without a history of HDP in the index pregnancy, a value of ≥ 1.0 kg/m^2^/year was associated with HDP in the subsequent pregnancy. However, it remains unclear whether HDP in the subsequent pregnancy can be prevented by controlling the annual BMI gain under these values.

Further research is needed to determine whether managing the annual BMI gain can prevent HDP in a subsequent pregnancy, which could reduce the risk of future CVD. Our study findings can be the basis for such research. Despite the challenge it presents, preventing HDP recurrence will improve the future health outcomes of women.

## Supplementary Information

Supplementary Information 1.

Supplementary Information 2.

Supplementary Information 3.
