# Supplementary material for: Annual body mass index gain and risk of hypertensive disorders of pregnancy in a subsequent pregnancy
Source: Sci Rep. 2021 Nov 18;11:22519. doi: 10.1038/s41598-021-01976-y (PMC8602630; doi:10.1038/s41598-021-01976-y)
Supplement: Supplementary file 2 — Supplementary Information 2. [file 41598_2021_1976_MOESM2_ESM.docx]

**Supplementary**

**Figure legends**

**Figure S1.** **Adjusted odds ratios for HDP in the subsequent pregnancy among those with a history of HDP in the index pregnancy.** The multivariable models were adjusted for maternal age in the index pregnancy, pregnancy interval, and classified annual BMI change. Values on the left side are expressed as log_10_OR in the upper panel and as log_2_OR in the lower panel. The right side of the graph shows the incidence of HDP in the subsequent pregnancy according to the degree of annual BMI change. The number of HDP in the subsequent pregnancy/total number is also shown according to the degree of annual BMI change. BMI, body mass index; CI, confidence interval; HDP, hypertensive disorders in pregnancy.
