# Supplementary figures and images for: Annual body mass index gain and risk of hypertensive disorders of pregnancy in a subsequent pregnancy
Source: Sci Rep. 2021 Nov 18;11:22519. doi: 10.1038/s41598-021-01976-y (PMC8602630; doi:10.1038/s41598-021-01976-y)

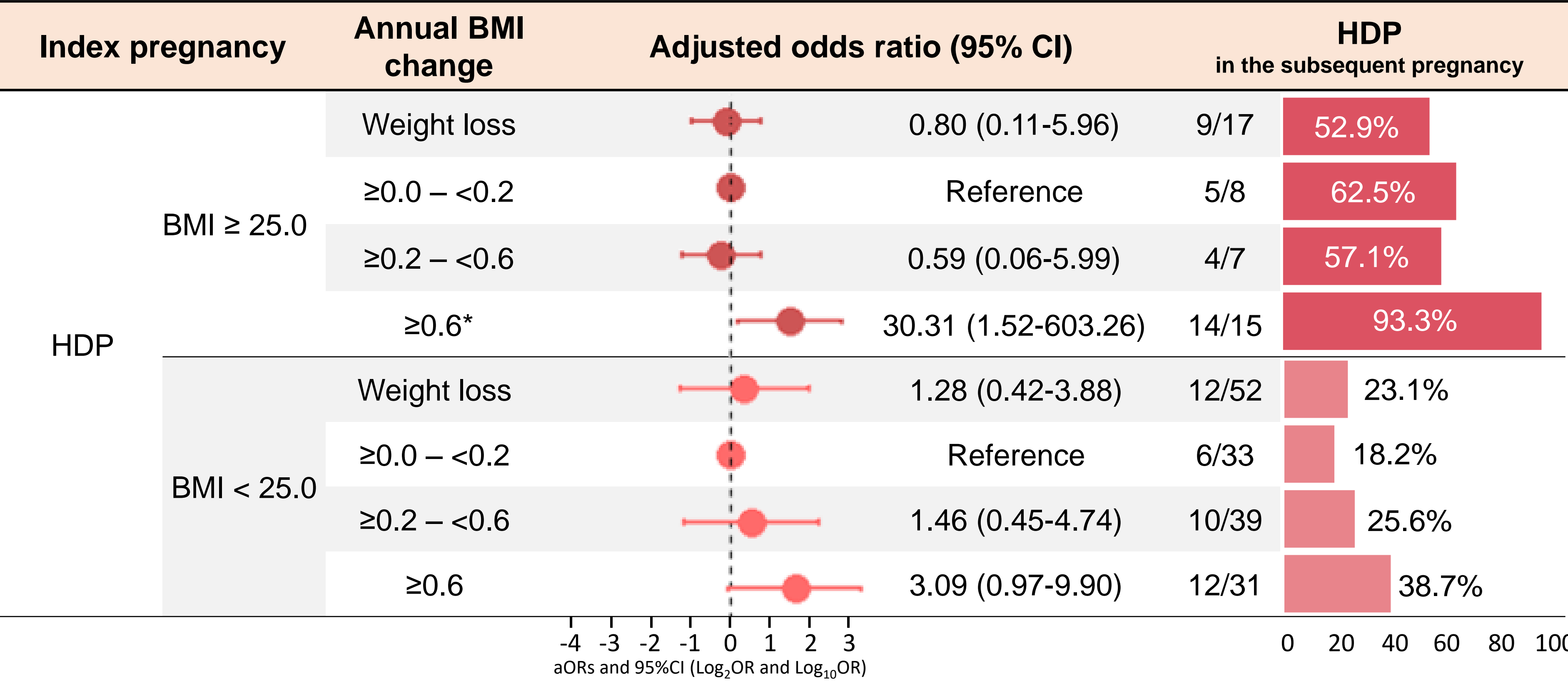

Supplement: Supplementary file 3 — Supplementary Information 3. [file 41598_2021_1976_MOESM3_ESM.pdf]
